# Tetracyclines: four rings to rule infections through resistance and disease tolerance

**DOI:** 10.1172/JCI162331

**Published:** 2022-09-01

**Authors:** Kátia Jesus, Luís F. Moita

**Affiliations:** Instituto Gulbenkian de Ciência, Oeiras, Portugal.

## Abstract

Several classes of antibiotics have long been known for protective properties that cannot be explained through their direct antimicrobial effects. However, the molecular bases of these beneficial roles have been elusive. In this issue of the *JCI*, Mottis et al. report that tetracyclines induced disease tolerance against influenza virus infection, expanding their protection potential beyond resistance and disease tolerance against bacterial infections. The authors dissociated tetracycline’s disease-resistance properties from its disease-tolerance properties by identifying potent tetracycline derivatives with minimal antimicrobial activity but increased capacity to induce an adaptive mitochondrial stress response that initiated disease tolerance mechanisms. These findings have potential clinical applications in viral infections.

## Strategies to survive severe infection

ICU patients with sepsis often present dramatically different outcomes despite having had a similar initiating pathogen or pathogen load, or even having completely eliminated the original infection. The contrasting outcomes may be explained by the need for two different but interdependent and evolutionarily conserved defense strategies to survive a severe infection: resistance, which relies on effector mechanisms to reduce pathogen load, and disease tolerance, which provides host tissue damage control and limits disease severity irrespective of pathogen load (1). Research on the initiation of protective immune responses has so far mostly focused on the direct sensing of microorganisms via pattern recognition receptors (PRRs). The pattern-triggered immunity model (2) states that PRRs recognize microorganism-associated molecular patterns (MAMPs) representative of different groups of microorganisms, which leads to the activation of effector mechanisms adjusted to each pathogen group. This model is well supported by data but fails to explain how the host can respond to pathogens with which it has no evolutionary history (3). Critically, the model is insufficient to explain how vertebrate hosts discriminate between commensal and pathogenic microorganisms that display similar MAMPs. While much progress has been made as to which and how immune circuits sense different groups of pathogens, current models still lack a comprehensive conceptual framework for immune responses. For example, the danger model (4) values contextual cues of pathogen-induced damage-associated molecular patterns but poorly explains the initiation step and has not resolved its mechanistic inconsistencies (5). Alternatively, the effector-triggered immunity (6) model proposes that the immune system recognizes pathogens by sensing virulence factors or activities (7, 8), but does not account for substantial cellular physiological perturbations that are not caused by the direct or indirect effects of virulence factors used by pathogens. It is likely that in addition to directly recognizing conserved microorganism molecular signatures using PRRs, the host mounts an immune response after sensing a homeostatic disruption that serves as a proximal reporter for infections (9). Interestingly, several groups of pathogens, including viruses and bacteria, target and perturb different organelles, including mitochondria (10). The host’s ability to sense a homeostatic disruption may be a key component for detecting the presence of a disease-causing microorganism. These pathways may synergize with the sensing capability of PRRs not only to potentiate the resulting feed-forward mechanisms that contribute to initiating the immune response, but also to inform the host on the intensity of the threat posed by the pathogen. The early events triggered by a disruption of homeostasis may also have a role in limiting tissue damage and in later negative-feedback pathways that terminate the inflammatory response and activate tissue repair, allowing for a full return to steady state.

## Antibiotics have effects beyond their direct antimicrobial activities

Physicians have known, and empirically used for decades, several classes of antibiotics that seem to better resolve an infection than would be expected from their direct antimicrobial efficacy alone, comparing favorably with other classes that have similar antimicrobial spectra. In addition, for example, macrolides have extensively documented clinically beneficial roles in chronic inflammatory pulmonary disorders (11, 12), and demonstrated protective effects in models of cerebral ischemia (13). Other classes, including fluoroquinolones and tetracyclines, have also been vaguely labeled as immunomodulators, but the molecular mechanistic bases remain unidentified (14). More recently, aminoglycosides were shown to enhance host resistance to viral infections independently of their antibacterial effects, in a microbiota-independent manner (15). These antiviral effects are based on an induced interferon (IFN) response and are, therefore, a surprising example of host resistance against viral infection that is enhanced by antibiotics.

Antibiotics, like most other drugs in clinical use, have varying degrees of off-target effects that may account for undesirable side effects. Unexpectedly, some of these off-target effects induce low-level core cellular function perturbations in the host, which may constitute a critical signal to initiate both resistance and disease tolerance mechanisms. For example, quinolones that target the bacterial enzymes DNA gyrase and DNA topoisomerase IV cause low levels of DNA damage to the host, potentially leading to the induction of IFN-stimulated genes (ISGs), as in the case of aminoglycoside antibiotics (15). Additionally, ribosome-targeting antibiotics (RAbos), which include tetracyclines, are bacteriostatic because they block bacterial ribosomes but also mildly inhibit host mitochondrial protein synthesis, given the similarity of the host mitoribosome and bacterial ribosomes. Tetracyclines are a group of broad-spectrum antibiotics that share a common chemical structure based on four (tetra-) linearly fused hydrocarbon rings. This tetracycline nucleus can be modified by the attachment of a diverse set of functional groups that shape their properties. They are active against a wide range of microorganisms that include Gram-positive and Gram-negative bacteria, chlamydiota, mycoplasmatota, rickettsiae, and protozoan parasites. They also have extensive applications outside infection, including in dermatologic conditions like acne. Our laboratory has recently found that host inhibition of mitochondrial protein synthesis by tetracycline antibiotics perturbs the electron transport chain (ETC), leading to improved damage repair in the lung in addition to adrenergic and glucocorticoid sensitivity in the liver. These findings explain the microbiome-independent induction of disease tolerance against sepsis models initiated by tetracycline-resistant bacterial infection (16).

## Tetracyclines induce disease tolerance against viral infection

**Figure 1:**
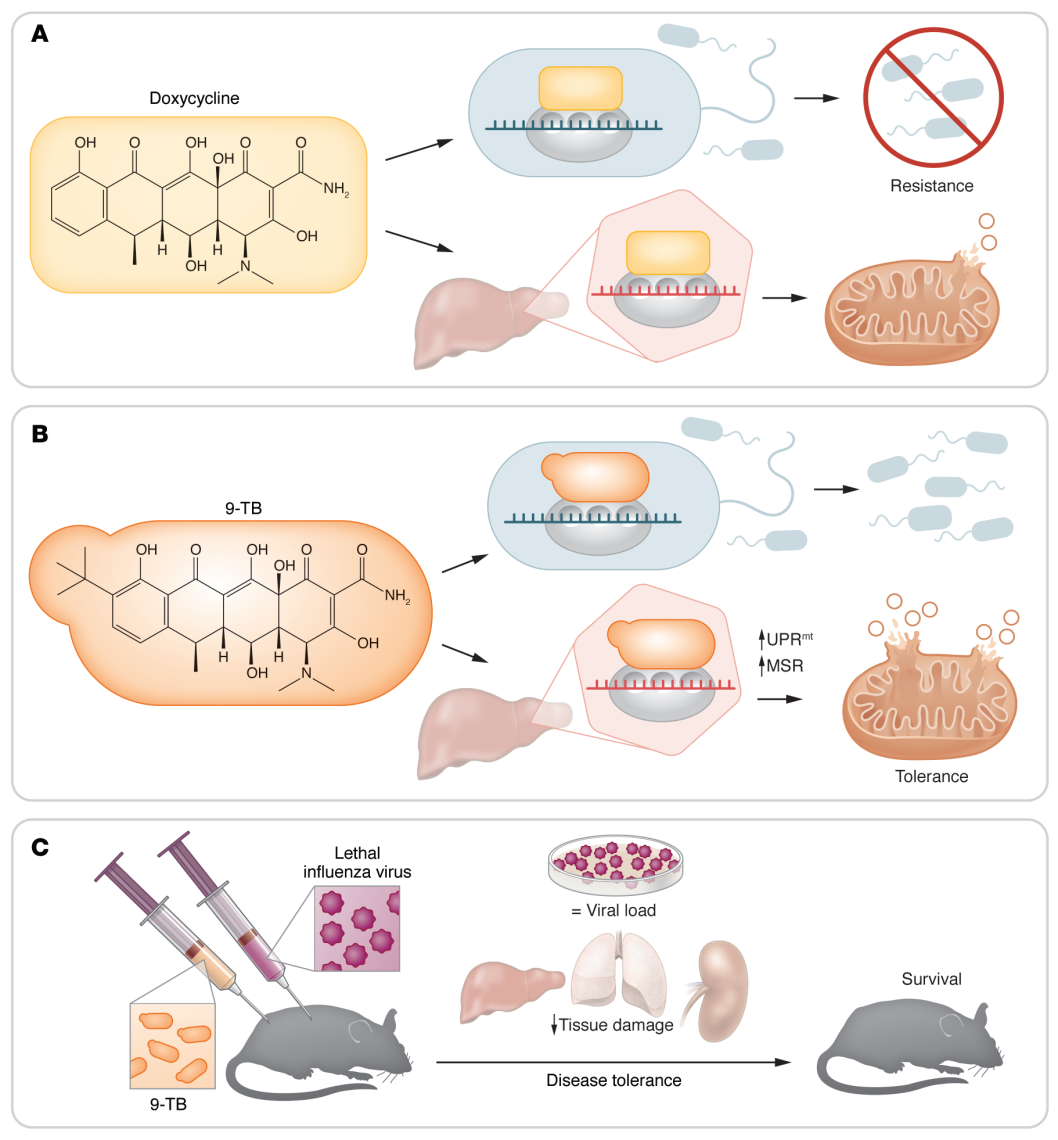
Tetracycline derivatives with minimal antimicrobial activity have increased capacity to induce an adaptive mitochondrial stress response and enhance disease tolerance. (**A**) Doxycycline, a prototypical tetracycline antibiotic, blocks bacterial and mitochondrial translation, inducing mild proteotoxic mitochondrial stress, which initiates mitochondrial stress responses. (**B**) 9-*tert*-Butyl doxycycline (9-TB), a doxycycline derivative with a substitution at the C9 position, has minimal antimicrobial activity but shows substantially greater capacity to induce the UPR^mt^ and mitochondrial stress response (MSR) when compared with parental doxycycline. (**C**) Mottis et al. (17) showed that both parental doxycycline and 9-TB improved survival of mice in a model of lethal influenza virus infection, by reducing tissue damage but without affecting viral titers. This finding demonstrates that in addition to the antimicrobial properties of tetracyclines (known as resistance), the effect of this class of antibiotics on the host mitochondria triggers disease tolerance mechanisms in viral infections through activation of MSRs.

In this issue of the *JCI*, Mottis and colleagues take these observations further to demonstrate that tetracyclines also induce disease tolerance (Figure 1) to influenza virus (IFV) infections (17). Using cellular and germ-free mouse models, where tetracyclines decrease oxidative phosphorylation complex activity and ATP concentrations, the authors demonstrated that the prototypical four-ringed tetracycline doxycycline caused a mild mitochondrial stress response that included both type I IFN signaling and an activating transcription factor 4–mediated (ATF4-mediated) integrated stress response (ISR). Doxycycline caused distinct transcriptional responses in the kidney and liver. While the kidney responded with a transcriptional signature pointing to the activation of the ATF4/ISR pathway, including characteristic translation inhibition, the liver induced a type I IFN response with increased expression of ISGs. Using bone marrow–derived macrophages (BMDMs), the authors identified the release of mtDNA from mitochondria, following its perturbation by doxycycline, as the likely trigger for the initiation of the type I IFN response.

To avoid the antibacterial effects of tetracyclines on the host microbiome, Mottis et al. (17) identified several derivatives with minimal antimicrobial activity. In particular, a derivative with a substitution at the C9 position, 9-*tert*-butyl doxycycline (9-TB), retained, and in fact substantially superseded, the effects of the parental doxycycline on induction of mitochondrial unfolded protein response (UPR^mt^). 9-TB was also much more potent than doxycycline at inducing a mitochondrial stress response (as measured by the capacity to affect mitonuclear protein imbalance) and inducing ISGs in BMDMs. Doxycycline and 9-TB were effective at increasing survival in a lethal IFV infection model when given preventively. They did not affect the viral titers, a fact that points to their capacity to induce disease tolerance, not resistance mechanisms. Critically, while doxycycline affected the gut microbiome as expected, decreasing its bacterial species diversity, 9-TB did not affect the microbiome composition. Moreover, 9-TB was also capable of decreasing the severity of infection and delaying mortality when administered therapeutically (Figure 1). The authors further showed that disease tolerance to IFV infection correlated with the induction of genes associated with lung epithelia and cilia function. In addition, 9-TB (to a greater extent than doxycycline) downregulated genes with roles in inflammatory and immune responses in the lung, liver, and kidney, possibly limiting tissue damage resulting from an excessive inflammatory response to infection. These findings agree with our demonstration that RAbos impair T cell effector function and ameliorate autoimmunity by blocking mitochondrial protein synthesis (18) because T cells may often cause collateral tissue damage.

Going forward, many exciting questions remain. One is how core cellular function perturbations leading to resistance or disease tolerance are sensed. In the case of tetracyclines it is tempting to speculate that inhibiting mitochondrial protein synthesis perturbs the ETC and decreases ATP concentration, potentially leading to the initiation of UPR^mt^. This possibility is based on the recent finding that ATP is a strong hydrotrope with the ability to prevent the formation of, and dissolve already formed, protein aggregates (19). Alternatively, tetracycline-induced inhibition of mitochondrial protein synthesis may cause an altered stoichiometry of the ETC complex components that are encoded by nuclei and mitochondria, constituting a signal that is sensed and transduced by unknown factors. A second category of questions will emerge from the systematic investigation of the types of infections that may benefit from the disease tolerance–inducing properties of tetracyclines. Different groups of pathogens impose specific types of tissue damage and are antagonized by appropriate nonoverlapping immune effector responses. Each one of these effector mechanisms comes with its own specific immunopathology and requires unique disease tolerance processes to resolve each pathogen-specific type of tissue damage. Of course, tetracyclines are just the tip of the iceberg. Many other classes of immunomodulatory antibiotics cause their own types of perturbations to cellular processes and organelles. The mechanistic study of their effects is likely to reveal fundamental biological insights into the regulation of organismal homeostasis by stress responses. This knowledge may allow us to harness antibiotic effects for therapeutic strategies against infection and other conditions that progress with inflammation and substantial tissue damage and loss of function, including autoimmune, neurodegenerative, and cardiovascular diseases.

## Conclusions and clinical implications

The study by Mottis et al. (17) substantially adds to our understanding of the mechanisms of tetracycline-induced disease tolerance and extends the effects to viral infections, potentially opening the possibility of using tetracycline derivatives as adjuvants for viral infection treatment. Critically, this work demonstrates that the antimicrobial (resistance) activity and the effects on host mitochondria (disease tolerance) of tetracyclines can be dissociated by the identification of potent tetracycline derivatives like 9-TB, which retain minimal antimicrobial activity but substantially increase effects on mitochondrial function with the potential to induce disease tolerance and minimize tissue damage.

## Version 1 09/01/2022

Electronic publication
